# Hydrogen Sulfide Donor GYY4137 Protects against Myocardial Fibrosis

**DOI:** 10.1155/2015/691070

**Published:** 2015-05-11

**Authors:** Guoliang Meng, Jinbiao Zhu, Yujiao Xiao, Zhengrong Huang, Yuqing Zhang, Xin Tang, Liping Xie, Yu Chen, Yongfeng Shao, Albert Ferro, Rui Wang, Philip K. Moore, Yong Ji

**Affiliations:** ^1^Key Laboratory of Cardiovascular Disease and Molecular Intervention, Atherosclerosis Research Centre, Nanjing Medical University, Nanjing 210029, China; ^2^Department of Pharmacology, School of Pharmacy, Nantong University, Nantong 226001, China; ^3^Aoyong Hospital, Zhangjiagang 215600, China; ^4^Department of Pathology, Jincheng People's Hospital, Jincheng 048000, China; ^5^Department of Cardiology, The First Affiliated Hospital of Xiamen University, Xiamen 361003, China; ^6^Department of Cardiology, The Affiliated Jiangning Hospital of Nanjing Medical University, Nanjing 211100, China; ^7^Department of Anesthesia, The First Affiliated Hospital of Nanjing Medical University, Nanjing 210029, China; ^8^Department of Thoracic and Cardiac Surgery, The First Affiliated Hospital of Nanjing Medical University, Nanjing 210029, China; ^9^Cardiovascular Division, Department of Clinical Pharmacology, British Heart Foundation Centre of Research Excellence, Faculty of Life Sciences and Medicine, King's College London, London SE1 9NH, UK; ^10^Department of Biology, Lakehead University, Thunder Bay, ON, Canada P7B 5E1; ^11^Department of Pharmacology, National University of Singapore, Singapore 117597

## Abstract

Hydrogen sulfide (H_2_S) is a gasotransmitter which regulates multiple cardiovascular functions. However, the precise roles of H_2_S in modulating myocardial fibrosis *in vivo* and cardiac fibroblast proliferation *in vitro* remain unclear. We investigated the effect of GYY4137, a slow-releasing H_2_S donor, on myocardial fibrosis. Spontaneously hypertensive rats (SHR) were administrated with GYY4137 by intraperitoneal injection daily for 4 weeks. GYY4137 decreased systolic blood pressure and inhibited myocardial fibrosis in SHR as evidenced by improved cardiac collagen volume fraction (CVF) in the left ventricle (LV), ratio of perivascular collagen area (PVCA) to lumen area (LA) in perivascular regions, reduced hydroxyproline concentration, collagen I and III mRNA expression, and cross-linked collagen. GYY4137 also inhibited angiotensin II- (Ang II-) induced neonatal rat cardiac fibroblast proliferation, reduced the number of fibroblasts in S phase, decreased collagen I and III mRNA expression and protein synthesis, attenuated oxidative stress, and suppressed *α*-smooth muscle actin (*α*-SMA), transforming growth factor-*β*1 (TGF-*β*1) expression as well as Smad2 phosphorylation. These results indicate that GYY4137 improves myocardial fibrosis perhaps by a mechanism involving inhibition of oxidative stress, blockade of the TGF-*β*1/Smad2 signaling pathway, and decrease in *α*-SMA expression in cardiac fibroblasts.

## 1. Introduction

About two-thirds of cells within the heart consist of nonmyocardial cells, including fibroblasts, smooth muscle cells, and endothelial cells, of which more than 90% are fibroblasts. Cardiac fibrosis, a common pathological process which occurs in the context of many different heart diseases, may increase myocardial stiffness, hamper ventricular diastolic and systolic function, and eventually trigger heart failure [1]. Excessive accumulation of collagen fibers in the heart is one of the main manifestations of myocardial fibrosis [2]. Methods for retarding cardiac fibrosis are rare but include treatment with some antihypertensive drugs [3, 4], inhibitors of matrix metalloproteinases [5], microRNA intervention [6], and stem cell transplantations [7]; however, none of these treatment provides satisfactory outcomes in the clinic.

Hydrogen sulfide (H_2_S), the third gasotransmitter discovered after nitric oxide and carbon monoxide, plays a key role in modulating cardiovascular function [8–10]. Much evidence now confirms a role for H_2_S in a wide range of physiological and pathological processes in the cardiovascular system, including blood pressure lowering, vasorelaxation, cardioprotection, and inhibition of atherosclerosis [11–15]. The effect of H_2_S on fibrosis has been studied previously by several groups. Using whole cell patch clamping, NaHS has been found to inhibit human atrial fibroblast proliferation induced by transforming growth factor-*β*1 (TGF-*β*1) or 20% fetal bovine serum (FBS). Preconditioning of fibroblasts with NaHS decreases basal expression of Kv4.3 (encode I(to)), but not KCa1.1 (encode BK(Ca)) and Kir2.1 (encode IK(ir)) [16]. Furthermore, H_2_S attenuates TGF-*β*1-stimulated Kv4.3 and *α*-smooth muscle actin (*α*-SMA) expression, in parallel with its ability to inhibit TGF-*β*-induced myofibroblast transformation [16]. Huang et al. reported that H_2_S suppressed cardiac fibrosis induced by pressure overload, possibly by inhibiting the activity of intracardiac angiotensin II (Ang II) and by modifying the expression of cx43 [17]. NaHS also inhibits Ang II-induced expression of *α*-SMA, connective tissue growth factor (CTGF), and type I collagen and additionally upregulates the expression of heme oxygenase-1 (HO-1), in cardiac fibroblasts [18].

Nevertheless, the pathogenesis of myocardial fibrosis is complex and the precise role of H_2_S in modulating myocardial fibrosis* in vivo *and cardiac fibroblast proliferation* in vitro* remains unclear. One reason for such lack of clarity to date may be the reliance on NaHS as H_2_S donor in previous studies. NaHS has been very widely used to evaluate the biology of H_2_S and has provided useful information about the pharmacological effects of this gas. However, NaHS releases copious amounts of H_2_S over a very short time frame (seconds) and as such is unlikely to mimic the time course of H_2_S release* in vivo* [19]. With this in mind, we have therefore now evaluated the biological effects in this system of GYY4137 (morpholin-4-ium-methoxyphenyl-morpholino-phosphinodithioate), which releases low concentrations of H_2_S slowly (hours) in aqueous solution at physiological pH and temperature [20]. Previous work with GYY4137 has revealed its ability to reduce systolic blood pressure (SBP) in spontaneously hypertensive rats (SHR) [20], highlighting a potential beneficial therapeutic effect on myocardial fibrosis, which is a common and important complication of hypertension.

The aims of the present study were therefore to examine whether GYY4137 is able to attenuate myocardial fibrosis in SHR* in vivo* and Ang II-induced cardiac fibroblast proliferation* in vitro* and to elucidate the mechanism of such effects. The present data raise the novel possibility that treatment with slow-releasing H_2_S donors can provide a novel therapeutic approach to reduce the development of myocardial fibrosis.

## 2. Materials and Methods

### 2.1. Treatment of Animals

Male SHR and Wistar-Kyoto (WKY) rats at 12 weeks of age were obtained from Shanghai SLAC Laboratory Animal Co., Ltd. (Shanghai, China). Animals were housed at constant temperature (20–22°C) and humidity (45–55%), with a 12 h light-dark cycle and fed a standard rat chow (SLAC) and water* ad libitum*. Following a 3-day acclimatization period, normotensive WKY rats served as controls (WKY group, *n* = 10) and age-matched SHRs were randomly divided into 4 groups (*n* = 10 per group) assigned different dosages of GYY4137: 0 (SHR group), 10 (GYY10 group), 25 (GYY25 group), or 50 (GYY50 group) mg/kg/d. GYY4137 was given by intraperitoneal injection once daily over a 4-week period. WKY and SHR control groups received the same volume of physiological saline instead of drug once daily over the same time period. During treatment, SBP was measured by the tail-cuff method with a Visitech BP-2000 blood pressure analysis system (Visitech Systems, Apex, NC, USA) under minimal restraint.

Animal experiments were performed in accordance with the NIH Guidelines for Care and Use of Laboratory Animals. This study was approved by the Committee on Animal Care of Nanjing Medical University.

### 2.2. Echocardiography

After treatment, cardiac systolic function was evaluated in pentobarbital-anesthetized rats using an echocardiography system (Visual Sonics Vevo 2100, VisualSonics, CA) equipped with a 12 MHz linear-array transducer. Two-dimensional (2D) images were obtained in the parasternal long-axis and short-axis views as well as apical two- and four-chamber views. Left ventricular (LV) ejection fraction (EF) and fractional shortening (FS) were derived by goal-directed, diagnostically driven software.

### 2.3. Histology

After 4 weeks of treatment as above, rats were anesthetized with pentobarbital and killed and the heart was excised immediately. The upper mid-level LV slices were dehydrated and embedded in paraffin. Two sequential 5-*μ*m-thick sections were obtained from each heart. The sections were stained with the collagen-specific picrosirius red (Sigma, St. Louis, MO). A total of 15 myocardial images from each slide were captured by light microscopy (Leica, Germany) and analyzed using the Image Pro Plus program (Media Cybernetics, Bethesda, MD, USA). For each image, the collagen volume fraction (CVF) in LV was determined as the ratio of collagen surface area to myocardial surface area. Quantitative evaluation of perivascular fibrosis was also performed, by imaging a field surrounding an intramyocardial artery and determining the ratio of perivascular collagen area (PVCA) to lumen area (LA).

### 2.4. Myocardial Collagen Measurement

Myocardial hydroxyproline concentration was determined as previously described [21]. Myocardial collagen was extracted and digested with cyanogen bromide (CNBr) according to the procedure described previously [22]. The remaining portion of the CNBr-digested collagen sample was subjected to acid hydrolysis and hydroxyproline was determined as a measure of non-cross-linked (soluble) collagen. The amount of cross-linked (insoluble) collagen in the myocardium was determined based on total myocardial collagen amount. The ratio between cross-linked and non-cross-linked collagen was taken as an index of the degree of collagen cross-linking [3].

### 2.5. Culture and Treatment of Cardiac Fibroblasts

Sprague-Dawley rats, 1–3 days old, were anesthetized with ether prior to euthanasia. Hearts were removed immediately and ventricles were separated from the atria, trisected, and digested completely with 0.25% trypsin (Beyotime, Haimen, China) at 37°C for 7–10 cycles. All supernatants from each cycle, except the first, were pooled. Dulbecco's modified Eagle's medium (DMEM, Wisent Inc., Canada) with 10% FBS (Wisent Inc., Canada) equal in volume to the supernatants was added to terminate digestion, and the mixture was centrifuged for 4.5 minutes at 2000 g. The cell pellet was resuspended in DMEM containing 10% FBS, 100 U/mL penicillin, and 100 *μ*g/mL streptomycin. Dispersed cells were incubated for 1.5 h in a 5% CO_2_ incubator. Nonmyocytes attached to the bottom of the dishes were subsequently incubated with DMEM supplemented with 10% FBS for an additional 2–4 days. Confluent cardiac fibroblasts (CFs) were treated with trypsin and subcultured. Subconfluent (>70% confluency) CFs grown in culture dishes from the second to third passages were used in all experiments. The medium was changed to DMEM supplemented with 0.5% FBS for 24 h. Confluent cells were preincubated with different concentrations of GYY4137 (12.5 *μ*M, 25 *μ*M, and 50 *μ*M) for 4 h, followed by Ang II (Sigma-Aldrich, St. Louis, MO; 10^−7^ M) stimulation for an additional 24 h. Culture medium without GYY4137 was used as a vehicle control.

### 2.6. Cell Count

Numbers of CFs were determined by cell counting Kit-8 (CCK-8, Beyotime, Shanghai, China), according to the manufacturer's directions.

### 2.7. Cell Cycle Analysis

After treatment as above mentioned, the CFs were harvested by trypsinization, washed in phosphate buffered saline (PBS), and resuspended in cold 70% ethanol. Finally, propidium iodide (20 *μ*g/mL) staining solution was added to the samples and cell cycles were analyzed on a flow cytometry (BD FACSCalibur, Ann Arbor, MI). Results were acquired from 10,000 cells.

### 2.8. Measurement of Hydroxyproline in Cell Culture Medium

After treatment, cell culture medium was collected for measuring hydroxyproline content according to the manufacturer's instructions (Beyotime, Shanghai, China).

### 2.9. Quantitative Real-Time Polymerase Chain Reaction

Quantitativereal-time PCR analysis was used to measure mRNA expression with 18S ribosomal RNA as a control. Total RNA was extracted from myocardium or CFs using Trizol reagent (Takara, Otsu, Shiga, Japan). RNA (500 ng) was added as a template to reverse-transcriptase reactions carried out using the PrimeScript RT Master Mix Kit (Takara, Otsu, Shiga, Japan). PCRs were carried out with the resulting cDNAs using the SYBR Green Premix (Takara, Otsu, Shiga, Japan) with ABI 7500 Real-Time PCR System (ABI, Carlsbad, CA). Experimental Ct values were normalized to 18S and relative mRNA expression was calculated versus a reference sample. Each sample was run and analyzed in triplicate. Primers used for amplification were collagen I: 5′-AGGGTCATCGTGGCTTCTCT-3′ and 5′-CAGGCTCTTGAGGGTAGTGT-3′; collagen III: 5′-AGCGGAGAATACTGGGTTGA-3′ and 5′-GATGTAATGTTCTGGGAGGC-3′; TGF-*β*1: 5′-GCCCTGGACACCAACTATTGC-3′ and 5′-GGAGCGCACGATCATGTTGG-3′; and 18S: 5′-AGTCCCTGCCCTTTGTACACA-3′ and 5′-GATCCGAGGGCCTCACTAAAC-3′.

### 2.10. Western Blotting Analysis

Protein samples were separated on sodium dodecyl sulfate polyacrylamide gel electrophoresis (SDS-PAGE) and transferred onto polyvinylidene fluoride (PVDF) membrane (Millipore, Billerica, MA). After blocking at room temperature in 5% w/v nonfat milk with TBST buffer (Tris-HCl 10 mM, NaCl 120 mM, and Tween-20 0.1%; pH 7.4) for 2 h, membranes were incubated overnight with the appropriate primary anti-collagen I, anti-collagen III, anti-TGF *β*1 (1 : 1000, Santa Cruz Biotechnology, Santa Cruz, CA), anti-Smad2, anti-p-Smad2 (1 : 500, Bioworld Technology, St. Louis Park, MN), or anti-GAPDH (1 : 6000, Sigma-Aldrich, St. Louis, MO) antibodies, at 4°C, followed by horseradish peroxidase- (HRP-) conjugated secondary antibody at room temperature for 2 h. Proteins were visualized by enhanced chemiluminescence substrate (ECL, Pierce, Rockford, IL).

### 2.11. Immunofluorescence Staining

After treatment, the cells were blocked with 10% bovine serum albumin (Solarbio, Beijing, China) and incubated with primary antibody against *α*-SMA (1 : 1000, Santa Cruz Biotechnology, CA), collagen I, collagen III, or negative IgG control for 16 h at 4°C. Immunoreactivity was visualized using Alexa Fluor 488 or Alexa Fluor 555 conjugated IgG (Beyotime, Haimen, China, 1 : 1000). Cells were counterstained with DAPI (5 *μ*g/mL, Beyotime, Haimen, China) and then evaluated under a fluorescence microscope (Nikon, Tokyo, Japan).

### 2.12. Measurement of Reactive Oxygen Species

Detection of intracellular reactive oxygen species (ROS) was accomplished by the use of a 2′,7′-dichlorofluorescein-diacetate (DCFH-DA, 10 *μ*M) liposoluble probe according to the manufacturer's instructions (Beyotime, Shanghai, China). This probe is hydrolyzed to 2′,7′-dichlorodihydrofluorescein (DCFH), which is available for oxidation by ROS to produce fluorescent 2′,7′-dichlorofluorescein (DCF). The fluorescence, with its intensity in proportion to the amount of ROS, was measured at 488 nm (excitation) and 528 nm (emission) by fluorescence microscopy (Nikon TE2000, Tokyo, Japan) and flow cytometry (BD FACSCalibur, Ann Arbor, MI).

### 2.13. Statistical Analysis

All data are expressed as mean ± standard error of mean (SEM) and analyzed using one-way ANOVA with Tukey's posttest analysis for comparison of intra- as well as intergroup variance. Statistical significance was taken when *P* < 0.05.

## 3. Results

### 3.1. GYY4137 Decreases Systolic Blood Pressure in SHR

**Figure 1:**
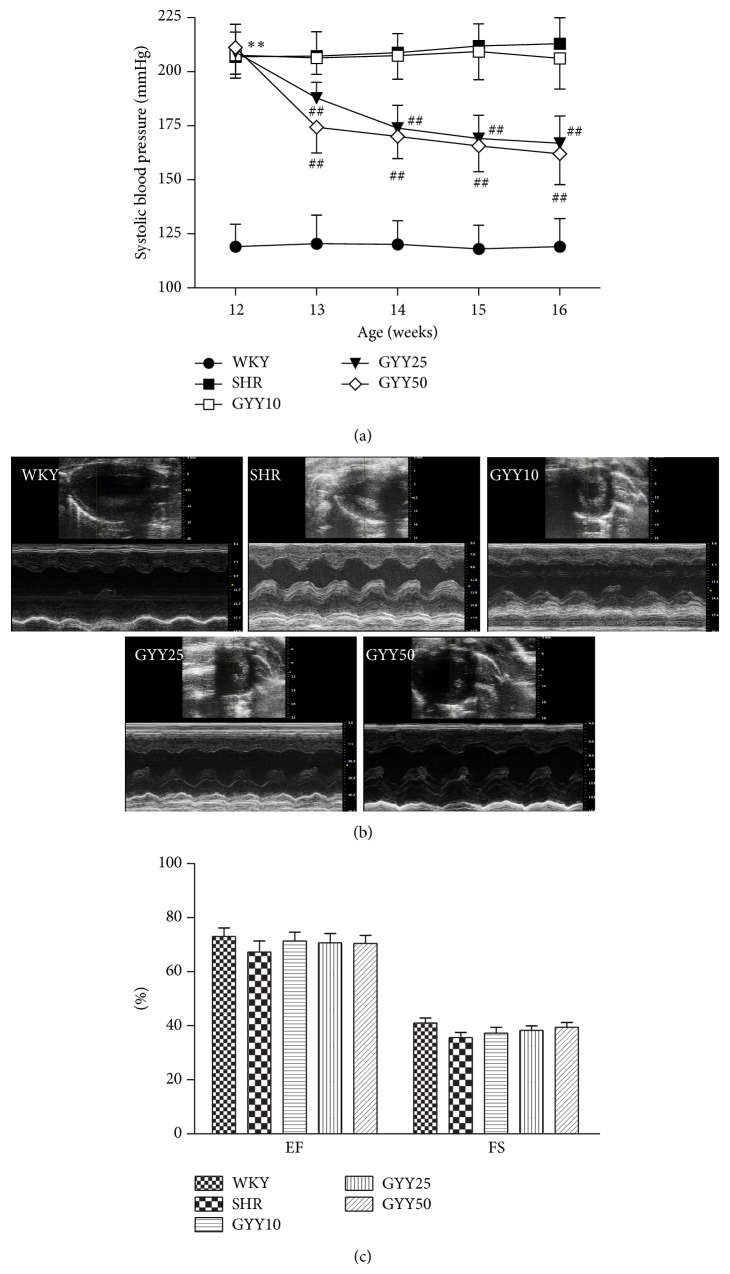
Effect of GYY4137 on systolic blood pressure and cardiac systolic function in SHR. Male SHR and WKY rats at 12 weeks of age were given GYY4137 by intraperitoneal injection at doses of 10 mg/kg/day (GYY10), 25 mg/kg/day (GYY25), or 50 mg/kg/day (GYY50) for 4 weeks. (a) Systolic blood pressure (SBP) was measured every week by the tail-cuff method. (b) Representative 2D M-mode echocardiograms in rat hearts after GYY4137 treatment for 4 weeks. (c) Quantitative analysis of left ventricular ejection fraction (EF) and fractional shortening (FS) with echocardiography. Plots represent mean ± SEM; *n* = 8–10. Statistical significance: ^**^ *P* < 0.01 compared with WKY; ^##^ *P* < 0.01 compared with SHR.

SHR at 12 weeks of age treated with GYY4137 for 4 weeks at 25 or 50 mg/kg/d, but not at 10 mg/kg/d, exhibited a significant reduction in SBP as measured by the tail-cuff method (Figure 1(a)). However, there was no significant difference of EF or FS between groups (Figures 1(b)-1(c)), suggesting that GYY4137 does not affect cardiac systolic function in SHR of this age and that the lowering of SBP by GYY4137 is likely not explained by changes in cardiac function.

### 3.2. GYY4137 Attenuates Myocardial Fibrosis in SHR

**Figure 2:**
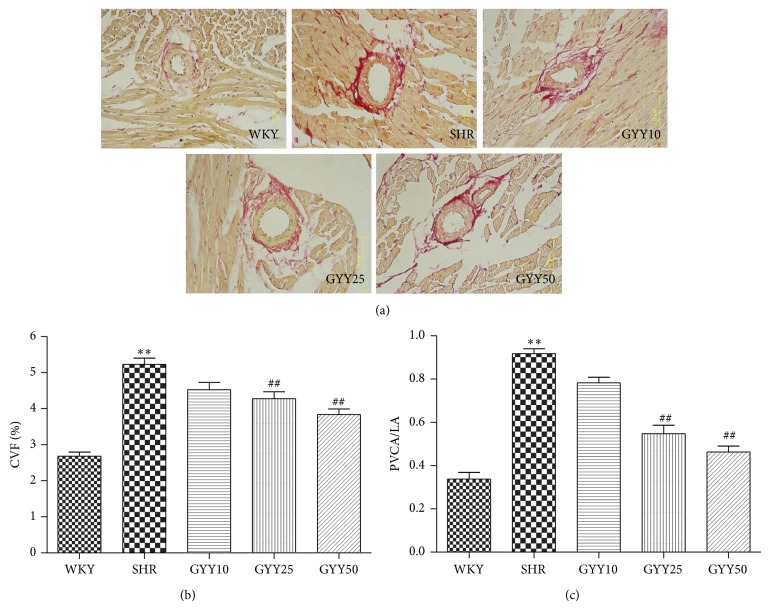
Effect of GYY4137 on myocardial fibrosis in SHR. Male SHR and WKY rats at 12 weeks of age were given GYY4137 by intraperitoneal injection at doses of 10 mg/kg/day (GYY10), 25 mg/kg/day (GYY25), or 50 mg/kg/day (GYY50) for 4 weeks. (a) Histological examination with picrosirius red staining of myocardium. (b) Collagen volume fraction (CVF) in left ventricle (LV) interstitial regions. (c) The ratio of perivascular collagen area (PVCA) to lumen area (LA) in perivascular regions. Plots represent mean ± SEM; *n* = 10. Statistical significance: ^**^ *P* < 0.01 compared with WKY; ^##^ *P* < 0.01 compared with SHR.

Picrosirius red stains myocardial cells yellow and collagen fiber red under light microscopy. A few of perivascular collagen fibers were seen in the myocardium of WKY rat, while a number of perivascular collagens accumulated along the myocardial interstitial matrix of SHRs. CVF and PVCA/LA ratio, two important indexes to evaluate the degree of myocardial interstitial and perivascular fibrosis, respectively, were quantitated. SHR exhibited increased CVF and PVCA/LA as compared to WKY rats (*P* < 0.01). GYY4137 (25 mg/kg/d and 50 mg/kg/d) treatment for 4 weeks caused a reduction in collagen-specific staining as well as CVF and PVCA/LA ratio (*P* < 0.01, Figure 2). Collectively these data show that GYY4137 efficiently attenuates myocardial fibrosis in SHR.

### 3.3. GYY4137 Improves Collagen Property in SHR

**Figure 3:**
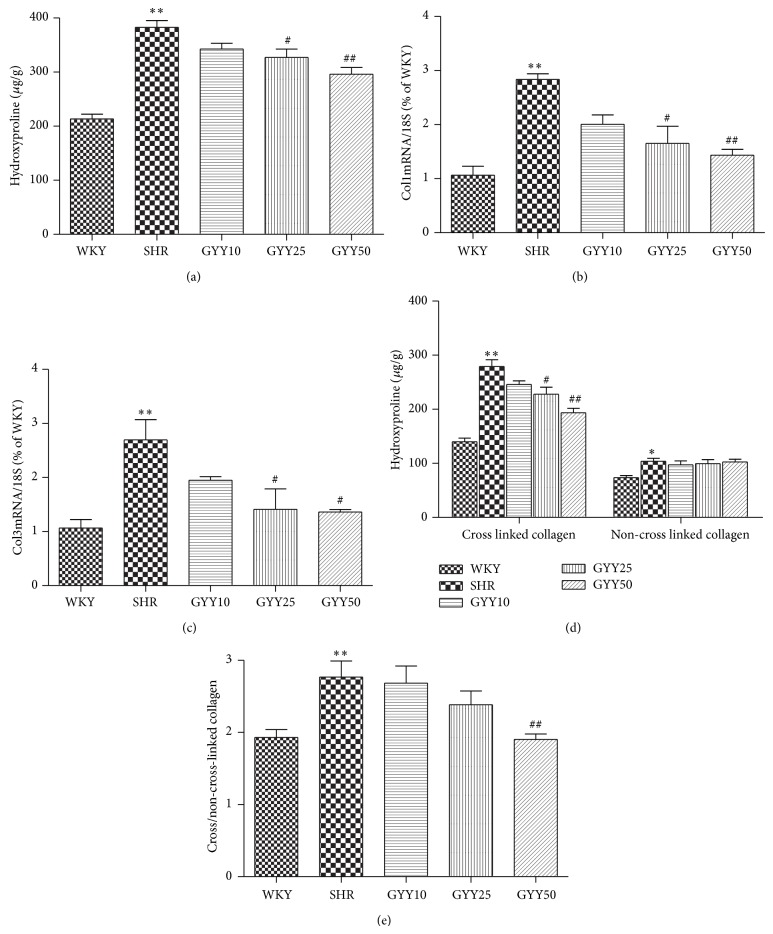
Effect of GYY4137 on collagen property in SHR. (a) Hydroxyproline concentrations in myocardium. (b-c) Quantification of collagen I and collagen III mRNA expression in myocardium was carried out with real-time RCR. (d) The amount of cross-linked and non-cross-linked collagen in the myocardium was evaluated as hydroxyproline content. (e) The ratio between cross-linked and non-cross-linked collagen was used as an index of the degree of collagen cross-linking. Plots represent mean ± SEM; *n* = 10. Statistical significance: ^*^ *P* < 0.05, ^**^ *P* < 0.01 compared with WKY; ^#^ *P* < 0.05, ^##^ *P* < 0.01 compared with SHR.

Total hydroxyproline content and expression of collagen I and III mRNA were higher in LV myocardium of SHR (*P* < 0.01), suggesting accumulation of collagen in SHR as compared to WKY rats. After GYY4137 administration at doses of 25 mg/kg/d or 50 mg/kg/d, both hydroxyproline content and collagen I and III mRNA expression in myocardium decreased (*P* < 0.05 and *P* < 0.01, Figures 3(a)–3(c)). SHR at 16 weeks of age exhibited an increase in both non-cross-linked (soluble) collagen and cross-linked (insoluble) collagen in myocardium (*P* < 0.01), and GYY 4137 at 25 mg/kg/d or 50 mg/kg/d evoked a decrease in cross-linked collagen without an increase in non-cross-linked collagen, and indeed, at the 50 mg/kg/d dosage, GYY4137 elicited a decline in the ratio of cross-linked to non-cross-linked collagen (*P* < 0.01, Figures 3(d)-3(e)).

### 3.4. GYY4137 Inhibits Ang II-Induced Cardiac Fibroblasts Proliferation

**Figure 4:**
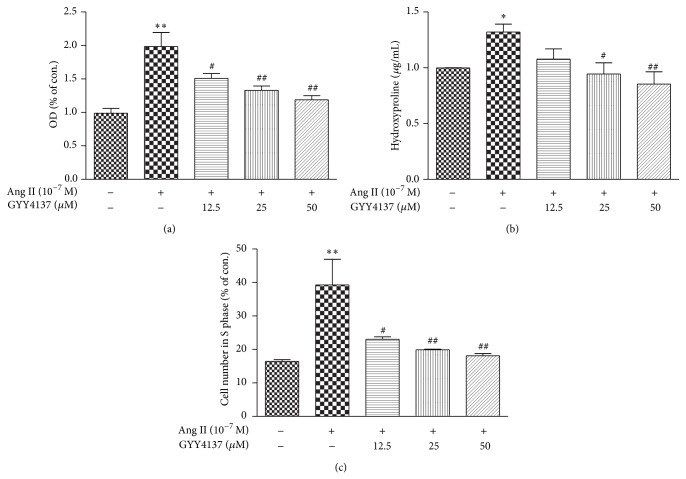
Effect of GYY4137 on Ang II-induced cardiac fibroblasts proliferation. Neonatal rat cardiac fibroblasts were pretreated with different concentrations of GYY4137 (12.5 *μ*M, 25 *μ*M, and 50 *μ*M) for 4 h followed by Ang II (10^−7^ M) stimulation for an additional 24 h. (a) The number of cells was represented as an OD value using a cell count assay. (b) Content of hydroxyproline in cell culture medium was determined. (c) Cell cycle was analyzed by flow cytometry. Plots represent mean ± SEM from 3–5 independent experiments. Cells treated with culture medium served as vehicle control (con.). Statistical significance: ^*^ *P* < 0.05, ^**^ *P* < 0.01, compared with con. group; ^#^ *P* < 0.05, ^##^ *P* < 0.01 compared with Ang II stimulation alone.

To determine whether GYY4137 inhibits CF proliferation, neonatal rat CFs were preincubated with different concentrations of GYY4137 for 4 h and then exposed to Ang II (10^−7^ M) for an additional 24 h. CF number was evaluated by cell count analysis (represented as an OD value) and content of hydroxyproline. Ang II increased CF number and hydroxyproline concentration in the medium, whilst preincubation with GYY4137 for 4 h inhibited CF proliferation (*P* < 0.05, Figures 4(a)-4(b)). Flow cytometry evaluation suggested that GYY4137 reduced the number of fibroblasts in the S phase of the cell cycle following Ang II-stimulation (*P* < 0.05, Figure 4(c)).

### 3.5. GYY4137 Reduces Ang II-Induced Collagen Synthesis in Cardiac Fibroblasts

**Figure 5:**
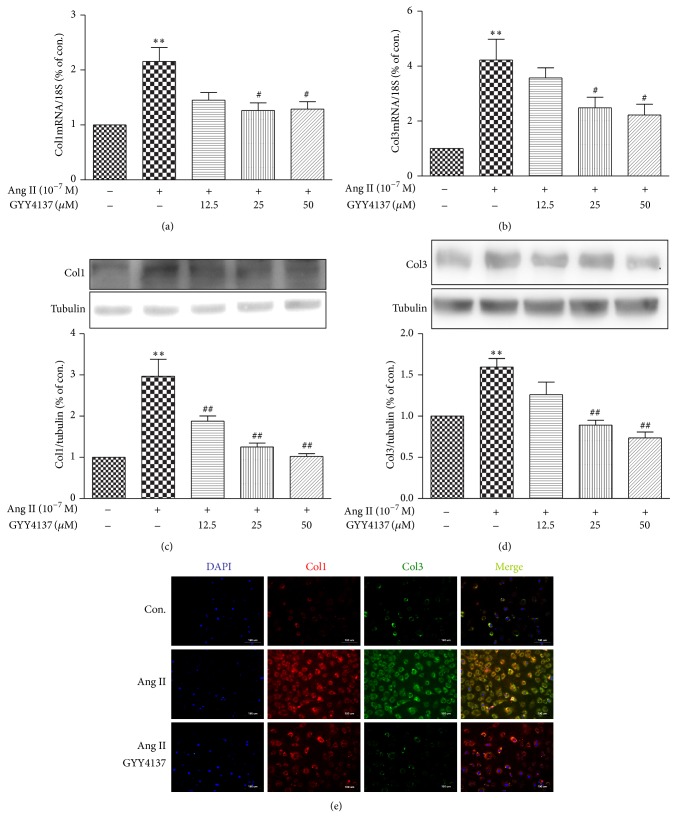
Effect of GYY4137 on Ang II-induced collagen synthesis in cardiac fibroblasts. Neonatal rat cardiac fibroblasts were pretreated with different concentrations of GYY4137 for 4 h followed by Ang II (10^−7^ M) stimulation for an additional 24 h. (a-b) Quantification of collagen I and collagen III mRNA expression in myocardium was carried out with real-time RCR. (c-d) Cell lysates were tested for collagen I and collagen III protein expression by western blotting. Tubulin was probed as a loading control. (e) Cellular collagen I and III were visualized using Alexa Fluor 555 or Alexa Fluor 488 conjugated IgG, respectively, by immunofluorescence staining. The nuclei were counterstained with DAPI (scale bar: 100 *μ*m). Plots represent mean ± SEM from 4–9 independent experiments. Cells treated with culture medium served as vehicle control (con). Statistical significance: ^**^ *P* < 0.01, compared with con. group; ^#^ *P* < 0.05, ^##^ *P* < 0.01 compared with Ang II stimulation alone.

Increase in collagen types I and type III is the predominant phenotype in cardiac fibrosis [23]. We therefore examined whether GYY4137 suppresses collagen synthesis after Ang II stimulation. Compared with medium-treated vehicle control, Ang II increased expression of collagen I and III at both the mRNA and protein levels, whose effect was attenuated by GYY4137 pretreatment (*P* < 0.05, Figures 5(a)–5(d)). Immunofluorescence staining was also carried out to further confirm that GYY4137 indeed inhibits collagen I and collagen III synthesis in Ang II-stimulated CFs (Figure 5(e)).

### 3.6. GYY4137 Blocks Ang II-Induced *α*-SMA and TGF-*β*1/Smad2 Expression in Cardiac Fibroblasts

**Figure 6:**
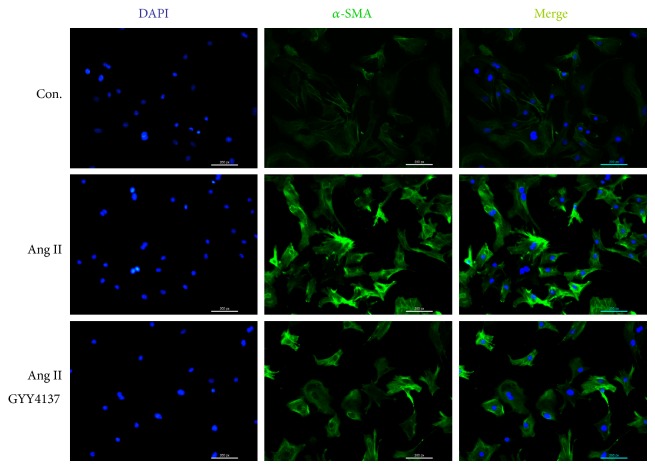
Effect of GYY4137 on *α*-SMA expression in cardiac fibroblasts. Neonatal rat cardiac fibroblasts were pretreated with different concentrations of GYY4137 for 4 h followed by Ang II (10^−7^ M) stimulation for an additional 24 h. Cellular *α*-SMA was visualized using Alexa Fluor 488 conjugated IgG by immunofluorescence staining. The nuclei were counterstained with DAPI (scale bar: 100 *μ*m).

Expression of *α*-SMA, one of the most robust markers of myofibroblast differentiation [24], was also measured by immunofluorescence. Expression of *α*-SMA was enhanced after Ang II stimulation, and this effect was attenuated by GYY4137 (50 *μ*M) pretreatment (Figure 6).

**Figure 7:**
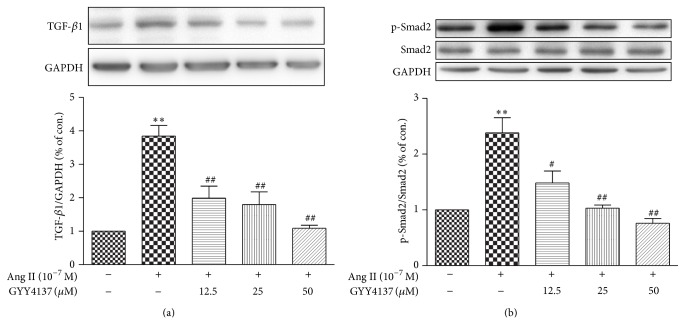
Effect of GYY4137 on TGF-*β*1/Smad2 expression in cardiac fibroblasts. Neonatal rat cardiac fibroblasts were pretreated with different concentrations of GYY4137 for 4 h followed by Ang II (10^−7^ M) stimulation for an additional 24 h. Cell lysates were tested for TGF-*β*1 (a) and Smad2 protein (b) expression by western blotting. GAPDH was probed as a loading control. Plots represent mean ± SEM from 4 independent experiments. Cells treated with culture medium served as vehicle control (con.). Statistical significance: ^**^ *P* < 0.01, compared with con. group; ^#^ *P* < 0.05, ^##^ *P* < 0.01 compared with Ang II stimulation alone.

TGF-*β*/Smad signal pathway facilitates the progression of myocardial fibrosis [25]. As expected, exposure of CFs to Ang II enhanced expression of TGF-*β*1 and phosphorylation of Smad2. Moreover, treatment with GYY4137 decreased TGF-*β*1 expression as well as Smad2 phosphorylation (*P* < 0.01, Figure 7).

### 3.7. GYY4137 Suppresses Ang II-Induced Oxidative Stress in Cardiac Fibroblasts

**Figure 8:**
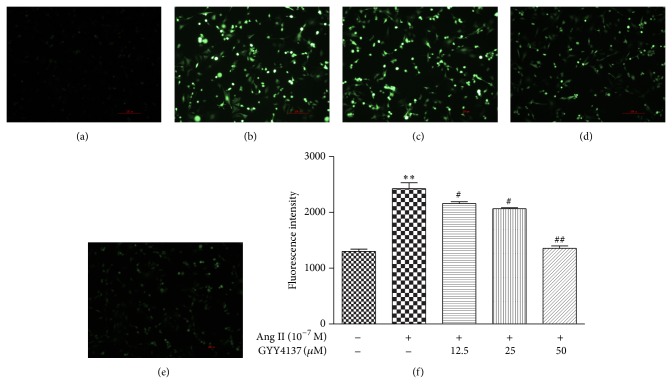
Effect of GYY4137 on Ang II-induced oxidative stress in cardiac fibroblasts. Neonatal rat cardiac fibroblasts were pretreated with different concentrations of GYY4137 for 4 h followed by Ang II (10^−7^ M) stimulation for an additional 24 h. (a–e) Levels of ROS in cardiomyocytes were measured by DCFH-DA (10 *μ*M) fluorescence staining (scale bar: 100 *μ*m). (a) Cells treated with culture medium served as vehicle control (con.); (b) Ang II stimulation alone; (c-d) cells pretreated GYY4137 (12.5 *μ*M, 25 *μ*M, and 50 *μ*M), respectively, for 4 h followed by Ang II (10^−7^ M) stimulation for an additional 24 h. (f) Fluorescence was estimated directly by flow cytometry. Plots represent mean ± SEM from 4 independent experiments. Cells treated with culture medium served as vehicle control (con.). Statistical significance: ^**^ *P* < 0.01, compared with con. group; ^#^ *P* < 0.05, ^##^ *P* < 0.01 compared with Ang II stimulation alone.

Compared with vehicle-treated control, Ang II induced severe oxidative stress in CFs as evidenced by increased intensity of DCFH fluorescence both on fluorescence microscopy and flow cytometry. These parameters were effectively restored by pretreatment with GYY4137 (*P* < 0.05, Figure 8).

## 4. Discussion

It has previously been reported that H_2_S protects against fibrosis in different systems. In a rat model of bleomycin-induced pulmonary fibrosis, the H_2_S donor diallyl sulfide attenuated excessive collagen production and extracellular matrix (ECM) protein expression [26]. In a rat model of unilateral ureteral obstruction, NaHS inhibited renal fibrosis by attenuating the production of collagen and of ECM, as well as the expression of *α*-SMA [27]. In mouse liver fibrosis induced by carbon tetrachloride, H_2_S blocked cell cycle activation and proliferation of stellate cells, thereby lessening liver fibrosis and reducing the accumulation of ECM [28]. Each of these examples suggests that H_2_S has antifibrotic effect in different tissues and organs. Moreover, Shi et al. found that chronic (3 months) NaHS treatment decreased interstitial fibrosis in SHR [29]. On the other hand, it is well established that NaHS promotes apoptotic cell death of cultured fibroblasts and smooth muscle cells and additionally releases copious amounts of H_2_S over a short time frame (seconds), which does not effectively mimic physiological concentrations of H_2_S* in vivo* and might be harmful [30]. However, previous studies have not evaluated specifically for possible toxicity of NaHS, and therefore whether the observed antifibrotic effect was simply a manifestation of H_2_S toxicity was unclear. Since NaHS is not an ideal H_2_S donor, several other H_2_S related compounds, including GYY4137 (a slow-releasing H_2_S donor), have been synthesized in order to better evaluate the physiological role(s) of H_2_S. When incubated in aqueous buffer (pH 7.4, 37°C), release of H_2_S from GYY4137 was slow. H_2_S increased for 15 minutes and then keeps a stabilized concentration up to 75 minutes. After administration (intravenous or intraperitoneal) of GYY4137 to anesthetized rats, plasma H_2_S concentration was increased at 30 minutes and remained elevated over the 180-minute time course of the experiment [20]. Moreover, GYY4137 did not cause detectable cytotoxicity or alter the cell cycle profile or p53 expression of cultured rat vascular smooth muscle cells; additionally, it did not trigger signaling pathways leading to cell death; and chronic treatment of conscious animals with GYY4137 at 133 *μ*mol/kg reduced SBP in SHR [20]. In the present work, daily GYY4137 administration for 4 weeks effectively reduced the degree of myocardial fibrosis in 16-week SHR as evidenced by collagen-specific staining. Not only total myocardial collagen amount but also perivascular fibrosis was also inhibited by GYY4137. These data demonstrate that GYY4137 has a powerful antifibrotic effect in the myocardium.

Excess collagen accumulation increases myocardial stiffness, impairs ventricular diastolic and systolic function, results in cardiac electrophysiological disorders, and eventually leads to heart failure; however, collagen deposition is also vital in maintaining myocardial structure and systolic heart function [31]. Therefore, it is important to study the details of alterations in collagen amount to comprehensively evaluate possible effects on myocardial fibrosis. Increase in myocardial cross-linked collagen, not simply total collagen, contributes to enhancement of stiffness, and the accumulation of cross-linked collagen decreases cardiac compliance in SHR. Cross-linked collagen is relatively resistant to degradation by proteases, which accelerates matrix accumulation [32]. In our study, increased accumulation of both cross-linked and non-cross-linked collagen was seen in myocardium of SHR; however, only cross-linked but not non-cross-linked collagen was affected by GYY4137; this might be expected to give rise to favourable functional effects in terms of reduced myocardial stiffness. This is the first study, to our knowledge, to demonstrate an inhibitory effect of H_2_S on cross-linked collagen content.

Fibroblasts are the main source of ECM during the process of myocardial fibrosis [33]. Ang II, one of the most important humoral factors which accelerate cardiac fibrosis, was used to stimulate CF proliferation. Fibrillar collagens, types I and III, are major structural proteins of the myocardial collagen matrix. Collagen type I, constituting 85% of total collagen, is a rigid protein (the tensile strength of collagen is 50–100 MPa and approaches that of steel) and is usually present in the form of thick fibers. The concentration of collagen type I determines the tissue stiffness of the myocardium. Collagen type III is more distensible and forms a fine reticular network. Myocardial fibrosis exhibits excess collagen and disarranged architecture in myocardium [34]. Our study found that Ang II increased numbers of fibroblasts as well as secretion of collagen, which were suppressed by GYY4137 administration. Moreover, levels of collagen types I and III were both attenuated by GYY4137.

It has been reported that NaHS increases the percentage of hepatic stellate cells (HSCs, the major cell type in hepatic fibrosis) in the G1 phase, whilst decreasing the percentage of cells in the S phase correspondingly [28]. NaHS also retards breast cancer cells transitioning from G1 to G0 phase and inhibits breast cancer cell division [35]. A 48 h treatment of diallyl sulfide induced G0/G1 cell cycle arrest in HeLa human cervical cancer cells [36]. These studies showed the potential ability of H_2_S to inhibit cell proliferation by regulating the cell cycle and hence DNA synthesis. In the present work, we found that cells in S phase increased after Ang II stimulation, which was rescued by GYY4137, indicating that GYY4137 might be able to inhibit cardiac fibroblast proliferation by reducing the number of cells in S phase.

Myofibroblasts have a particular ultrastructure between smooth muscle cells and fibroblasts, manifesting characteristics of both. Compared with ordinary fibroblasts, myofibroblasts, as the main source of ECM deposition, have greater ability to proliferate and secrete collagen [37]. *α*-SMA is one of the most robust markers of myofibroblast differentiation [38]. Previous studies have confirmed that H_2_S can inhibit the expression of *α*-SMA in obstructive nephropathy-induced renal fibrosis and carbon tetrachloride-induced liver fibrosis [27, 39]. Moreover, H_2_S attenuates TGF-*β*1-stimulated human atrial fibroblast proliferation via moderating their differentiation towards myofibroblasts [16]. Notably, in our study, *α*-SMA was reduced by GYY4137, suggesting that inhibition of fibroblast conversion into myofibroblasts by H_2_S may act as a vital mechanism to antagonize Ang II-induced profibrotic effects in CFs.

The molecular mechanisms regulating myocardial fibrosis are complex and as yet incompletely defined. Increasing evidence suggests that TGF-*β*1 signaling pathways play an important role in collagen synthesis [40]. It has been shown that H_2_S regulates the TGF-*β*1 pathway in multiple cell lines and tissues. For example, supplementation with H_2_S attenuates high-glucose-induced renal mesangial cells proliferation rate and production of TGF-*β*1 [41]. NaHS treatment also attenuates TGF-*β*1 levels in unilateral ureteral obstruction-induced kidney fibrosis in mice [42]. In the liver fibrosis model induced by carbon tetrachloride, exogenous H_2_S inhibits the expression of TGF-*β*1 and improves the liver fibrosis [28]. Moreover, NaHS treatment for 9 weeks prevented myocardial collagen remodeling in SHR by a mechanism involving inhibition of collagen synthesis via TGF-*β*1/Smad2/3 signaling pathway [43]. Our data show that GYY4137 inhibits the expression of TGF-*β*1 and Smad2 phosphorylation in Ang II-stimulated CFs. This action might therefore be responsible, at least in part, for the attenuation of cardiac fibroblast proliferation.

H_2_S may exert cardioprotection via its antioxidative effects. Thus, H_2_S protects against from myocardial ischemia/reperfusion injury by reducing ROS production and accumulation in the myocardium [44]. H_2_S also suppresses high glucose-induced cardiomyocyte apoptosis by attenuating ROS generation [45, 46]. Redox-sensitive signaling pathways play an important role in myocardial fibrosis and tempol (a powerful antioxidant) attenuates fibrosis in a ROS-inhibition-dependent manner in the renal fibrosis model induced by unilateral ureteral ligation [47]. Another study demonstrated that H_2_S was protective against gentamicin-induced nephrotoxicity in rats due to its antioxidant effect [48]. H_2_S also ameliorated cardiac fibrosis by decreasing oxidative stress in chronic heart failure [49]. However, the detailed mechanism by which H_2_S exerts its antioxidative effects is unclear. H_2_S can elicit vasoprotection by both scavenging O_2_^−^ and reducing vascular NADPH oxidase-derived O_2_^−^ production, in an acute oxidative stress model with xanthine oxidase or with the O_2_^−^ generator pyrogallol [50]. H_2_S has also been found to inhibit H_2_O_2_-mediated mitochondrial dysfunction in human endothelial cells by preserving the activities and protein expression levels of the antioxidant enzymes superoxide dismutase, catalase, glutathione peroxidase, and glutathione-S-transferase [51]. H_2_S also exerts neuroprotective effect by enhancing uncoupling protein 2-mediated antioxidation and subsequently suppressing ROS-triggered endoplasmic reticular stress [52]. H_2_S increases the nuclear localization of NF-E2 related factor 2 (Nrf2), a transcription factor that regulates the gene expression of a number of antioxidants (including heme oxygenase-1 and thioredoxin 1) during myocardial ischemia/reperfusion injury and renal interstitial fibrosis in diabetic rats [53, 54]. In accordance with these studies, our data demonstrate that the protective effect of GYY4137 against cardiac fibrosis depends, at least partly, on its antioxidant ability. However, similar to other H_2_S donors, the antioxidative effect of GYY4137 might be related to both direct scavenging activity and indirect regulation of antioxidant enzymes expression.

There are some limitations to our study. As stated above, the detailed antioxidative mechanism of GYY4137 in the context of myocardial fibrosis and Ang II-stimulated cardiac fibroblast proliferation is unclear and needs to be elucidated further. In addition, whether GYY4137 regulates ECM degradation, another important process in myocardial fibrosis, is not known. How GYY4137 decreases the expression of TGF-*β*1 also needs to be better defined.

In conclusion, we provide evidence that GYY4137 decreases myocardial fibrosis, which may be related to inhibition of oxidative stress, blockage of TGF-*β*1/Smad2 signaling pathway, and decrease in expression of *α*-SMA in cardiac fibroblasts. The present data raise the possibility that H_2_S may be of value in the treatment of cardiac fibrosis and related cardiovascular diseases which are underpinned by oxidative stress.

## References

[1] Kong P., Christia P., Frangogiannis N. G. (2014). The pathogenesis of cardiac fibrosis. *Cellular and Molecular Life Sciences*.

[2] Kwak H. B. (2013). Aging, exercise, and extracellular matrix in the heart. *Journal of Exercise Rehabilitation*.

[3] Meng G., Wu F., Yang L. (2009). Synergistic attenuation of myocardial fibrosis in spontaneously hypertensive rats by joint treatment with benazepril and candesartan. *Journal of Cardiovascular Pharmacology*.

[4] Roubille F., Busseuil D., Merlet N., Kritikou E. A., Rhéaume E., Tardif J.-C. (2014). Investigational drugs targeting cardiac fibrosis. *Expert Review of Cardiovascular Therapy*.

[5] Mukherjee R., Akar J. G., Wharton J. M. (2013). Plasma profiles of matrix metalloproteinases and tissue inhibitors of the metalloproteinases predict recurrence of atrial fibrillation following cardioversion. *Journal of Cardiovascular Translational Research*.

[6] Wijnen W. J., Pinto Y. M., Creemers E. E. (2013). The therapeutic potential of miRNAs in cardiac fibrosis: where do we stand?. *Journal of Cardiovascular Translational Research*.

[7] Elnakish M. T., Kuppusamy P., Khan M. (2013). Stem cell transplantation as a therapy for cardiac fibrosis. *Journal of Pathology*.

[8] Mani S., Untereiner A., Wu L., Wang R. (2014). Hydrogen sulfide and the pathogenesis of atherosclerosis. *Antioxidants & Redox Signaling*.

[9] Wang R. (2012). Physiological implications of hydrogen sulfide: a whiff exploration that blossomed. *Physiological Reviews*.

[10] Wang R. (2011). Signaling pathways for the vascular effects of hydrogen sulfide. *Current Opinion in Nephrology and Hypertension*.

[11] Chunyu Z., Junbao D., Dingfang B., Hui Y., Xiuying T., Chaoshu T. (2003). The regulatory effect of hydrogen sulfide on hypoxic pulmonary hypertension in rats. *Biochemical and Biophysical Research Communications*.

[12] Wang Y., Zhao X., Jin H. (2009). Role of hydrogen sulfide in the development of atherosclerotic lesions in apolipoprotein e knockout mice. *Arteriosclerosis, Thrombosis, and Vascular Biology*.

[13] Bos E. M., van Goor H., Joles J. A., Whiteman M., Leuvenink H. G. D. (2014). Hydrogen sulfide—physiological properties and therapeutic potential in ischaemia. *British Journal of Pharmacology*.

[14] Yang G., Zhao K., Ju Y. (2013). Hydrogen sulfide protects against cellular senescence via s-sulfhydration of keap1 and activation of Nrf2. *Antioxidants & Redox Signaling*.

[15] Tang G., Yang G., Jiang B., Ju Y., Wu L., Wang R. (2013). H_2_S is an endothelium-derived hyperpolarizing factor. *Antioxidants & Redox Signaling*.

[16] Sheng J., Shim W., Wei H. (2013). Hydrogen sulphide suppresses human atrial fibroblast proliferation and transformation to myofibroblasts. *Journal of Cellular and Molecular Medicine*.

[17] Huang J., Wang D., Zheng J., Huang X., Jin H. (2012). Hydrogen sulfide attenuates cardiac hypertrophy and fibrosis induced by abdominal aortic coarctation in rats. *Molecular Medicine Reports*.

[18] Pan L. L., Liu X. H., Shen Y. Q. (2013). Inhibition of NADPH oxidase 4-related signaling by sodium hydrosulfide attenuates myocardial fibrotic response. *International Journal of Cardiology*.

[19] Meng G., Ma Y., Ferro A., Ji Y. (2014). Emerging role of hydrogen sulfide in hypertension and related cardiovascular diseases. *British Journal of Pharmacology*.

[20] Li L., Whiteman M., Guan Y. Y. (2008). Characterization of a novel, water-soluble hydrogen sulfide-releasing molecule (GYY4137): new insights into the biology of hydrogen sulfide. *Circulation*.

[21] Stegemann H., Stalder K. (1967). Determination of hydroxyproline. *Clinica Chimica Acta*.

[22] Mukherjee D., Sen S. (1990). Collagen phenotypes during development and regression of myocardial hypertrophy in spontaneously hypertensive rats. *Circulation Research*.

[23] Sun Y., Weber K. T. (2005). Animal models of cardiac fibrosis. *Methods in Molecular Medicine*.

[24] Bai J., Zhang N., Hua Y. (2013). Metformin inhibits angiotensin II-induced differentiation of cardiac fibroblasts into myofibroblasts. *PloS ONE*.

[25] Weber K. T., Sun Y., Bhattacharya S. K., Ahokas R. A., Gerling I. C. (2013). Myofibroblast-mediated mechanisms of pathological remodelling of the heart. *Nature Reviews Cardiology*.

[26] Kalayarasan S., Sriram N., Soumyakrishnan S., Sudhandiran G. (2013). Diallylsulfide attenuates excessive collagen production and apoptosis in a rat model of bleomycin induced pulmonary fibrosis through the involvement of protease activated receptor-2. *Toxicology and Applied Pharmacology*.

[27] Song K., Wang F., Li Q. (2014). Hydrogen sulfide inhibits the renal fibrosis of obstructive nephropathy. *Kidney international*.

[28] Fan H.-N., Wang H.-J., Ren L. (2013). Decreased expression of p38 MAPK mediates protective effects of hydrogen sulfide on hepatic fibrosis. *European Review for Medical and Pharmacological Sciences*.

[29] Shi Y.-X., Chen Y., Zhu Y.-Z. (2007). Chronic sodium hydrosulfide treatment decreases medial thickening of intramyocardial coronary arterioles, interstitial fibrosis, and ROS production in spontaneously hypertensive rats. *American Journal of Physiology—Heart and Circulatory Physiology*.

[30] Baskar R., Li L., Moore P. K. (2007). Hydrogen sulfide-induces DNA damage and changes in apoptotic gene expression in human lung fibroblast cells. *The FASEB Journal*.

[31] Li A.-H., Liu P. P., Villarreal F. J., Garcia R. A. (2014). Dynamic changes in myocardial matrix and relevance to disease: translational perspectives. *Circulation Research*.

[32] de Haas H. J., Arbustini E., Fuster V., Kramer C. M., Narula J. (2014). Molecular imaging of the cardiac extracellular matrix. *Circulation Research*.

[33] de Jong S., van Veen T. A. B., de Bakker J. M. T., Vos M. A., van Rijen H. V. M. (2011). Biomarkers of myocardial fibrosis. *Journal of Cardiovascular Pharmacology*.

[34] Medugorac I., Jacob R. (1983). Characterisation of left ventricular collagen in the rat. *Cardiovascular Research*.

[35] Lv M., Li Y., Ji M.-H., Zhuang M., Tang J.-H. (2014). Inhibition of invasion and epithelial-mesenchymal transition of human breast cancer cells by hydrogen sulfide through decreased phospho-p38 expression. *Molecular Medicine Reports*.

[36] Wu P.-P., Chung H.-W., Liu K.-C. (2011). Diallyl sulfide induces cell cycle arrest and apoptosis in HeLa human cervical cancer cells through the p53, caspase- and mitochondria-dependent pathways. *International Journal of Oncology*.

[37] Kramann R., Dirocco D. P., Humphreys B. D. (2013). Understanding the origin, activation and regulation of matrix-producing myofibroblasts for treatment of fibrotic disease. *The Journal of Pathology*.

[38] Petschnik A. E., Fell B., Kruse C., Danner S. (2010). The role of *α*-smooth muscle actin in myogenic differentiation of human glandular stem cells and their potential for smooth muscle cell replacement therapies. *Expert Opinion on Biological Therapy*.

[39] Tan G., Pan S., Li J. (2011). Hydrogen sulfide attenuates carbon tetrachloride-induced hepatotoxicity, liver cirrhosis and portal hypertension in rats. *PLoS ONE*.

[40] Brik M., Santacruz B., Bancalari E. (2014). Posterior diaphragm agenesis: when liver simulates lungs. *Journal of Obstetrics and Gynaecology*.

[41] Xue H., Yuan P., Ni J. (2013). H_2_S inhibits hyperglycemia-induced intrarenal renin-angiotensin system activation via attenuation of reactive oxygen species generation. *PLoS ONE*.

[42] Jung K.-J., Jang H.-S., Kim J. I., Han S. J., Park J.-W., Park K. M. (2013). Involvement of hydrogen sulfide and homocysteine transsulfuration pathway in the progression of kidney fibrosis after ureteral obstruction. *Biochimica et Biophysica Acta*.

[43] Sun L., Jin H., Chen S. (2014). Hydrogen sulfide alleviates myocardial collagen remodeling in association with inhibition of TGF-beta smad signaling pathway in spontaneously hypertensive rats. *Molecular Medicine*.

[44] Peake B. F., Nicholson C. K., Lambert J. P. (2013). Hydrogen sulfide preconditions the db/db diabetic mouse heart against ischemia-reperfusion injury by activating Nrf2 signaling in an Erk-dependent manner. *The American Journal of Physiology–Heart and Circulatory Physiology*.

[45] Xu W., Wu W., Chen J. (2013). Exogenous hydrogen sulfide protects H9c2 cardiac cells against high glucose-induced injury by inhibiting the activities of the p38 MAPK and ERK1/2 pathways. *International Journal of Molecular Medicine*.

[46] Kuo W. W., Wang W. J., Tsai C. Y., Way C. L., Hsu H. H., Chen L. M. (2013). Diallyl trisufide (DATS) suppresses high glucose-induced cardiomyocyte apoptosis by inhibiting JNK/NFkappaB signaling via attenuating ROS generation. *International Journal of Cardiology*.

[47] Yoon H. E., Kim S. J., Chung S., Shin S. J. (2014). Tempol attenuates renal fibrosis in mice with unilateral ureteral obstruction: the role of PI3K-Akt-FoxO3a signaling. *Journal of Korean Medical Science*.

[48] Otunctemur A., Ozbek E., Dursun M. (2014). Protective effect of hydrogen sulfide on gentamicin-induced renal injury. *Renal failure*.

[49] Mishra P. K., Tyagi N., Sen U., Givvimani S., Tyagi S. C. (2010). H_2_S ameliorates oxidative and proteolytic stresses and protects the heart against adverse remodeling in chronic heart failure. *American Journal of Physiology—Heart and Circulatory Physiology*.

[50] Al-Magableh M. R., Kemp-Harper B. K., Ng H. H., Miller A. A., Hart J. L. (2014). Hydrogen sulfide protects endothelial nitric oxide function under conditions of acute oxidative stress in vitro. *Naunyn-Schmiedeberg's Archives of Pharmacology*.

[51] Wen Y.-D., Wang H., Kho S.-H. (2013). Hydrogen sulfide protects HUVECs against hydrogen peroxide induced mitochondrial dysfunction and oxidative stress. *PLoS ONE*.

[52] Lu M., Zhao F.-F., Tang J.-J. (2012). The neuroprotection of hydrogen sulfide against MPTP-induced dopaminergic neuron degeneration involves uncoupling protein 2 rather than ATP-sensitive potassium channels. *Antioxidants & Redox Signaling*.

[53] Zhou X., Feng Y., Zhan Z., Chen J. (2014). Hydrogen sulfide alleviates diabetic nephropathy in a streptozotocin-induced diabetic rat model. *The Journal of Biological Chemistry*.

[54] Calvert J. W., Jha S., Gundewar S. (2009). Hydrogen sulfide mediates cardioprotection through Nrf2 signaling. *Circulation Research*.

